# Adolescent delinquency following co-occurring childhood head injuries and conduct problem symptoms: findings from a UK longitudinal birth cohort

**DOI:** 10.1007/s00787-023-02335-0

**Published:** 2023-12-28

**Authors:** Hannah R. Carr, James E. Hall, Valerie C. Brandt

**Affiliations:** 1 School of Psychology, Centre for Innovation in Mental Health, University of Southampton, University Road, Highfield Campus, Building 44, Southampton, SO17 1PS UK; 2 Southampton Education School, University of Southampton, Southampton, UK; 3 Clinic of Psychiatry, Social Psychiatry and Psychotherapy, Hannover Medical School, Hanover, Germany

**Keywords:** Adolescence, Conduct problems, Delinquency, Head injury, Developmental psychopathology

## Abstract

Childhood conduct problems and head injuries share a bidirectional association, but how this affects the risk of adolescent delinquency is unknown. Due to their similar underlying mechanisms (i.e. increased impulsivity), this study aims to identify whether their co-occurrence increases the risk of adolescent delinquency. Data was obtained from 11,272 children at age 14 and 10,244 at age 17 years enrolled in the UK Millennium Cohort Study. Conduct problem symptoms (via the Strengths and Difficulties Questionnaire) and head injuries were parent reported from ages 3 to 14 years. Delinquency was self-reported at ages 14 and 17 including substance use, criminality, and antisocial behaviour. Incident rate ratios (IRR) were estimated for delinquency at ages 14 and 17 by childhood conduct problem and head injury status. Co-occurring head injuries and high conduct problem symptoms presented the greatest risk for overall delinquency and substance use at age 14 compared to those with the presence of one or neither (IRRs from 1.20 to 1.60). At age 17, conduct problems (with or without co-occurring head injuries) presented the greatest risk for overall delinquency, substance use, and antisocial behaviour. There was no evidence for an increased risk of delinquency at ages 14 or 17 following a head injury only. Whilst these findings suggest childhood head injuries alone do not increase the risk of adolescent delinquency, when co-occurring alongside high conduct problem symptoms there is a heightened earlier risk. These results provide further insight into adolescent delinquency and the outcomes of co-occurring childhood head injury and conduct problem symptoms.

**Supplementary Information:**

The online version contains supplementary material available at 10.1007/s00787-023-02335-0.

## Introduction

The adolescent stage of development is a crucial milestone for the maturation of social, emotional, and cognitive abilities. Adolescent delinquency can disrupt this critical phase of development leaving an individual vulnerable to a plethora of negative outcomes. Adolescent delinquency can include criminality, substance use, and antisocial behaviour. These behaviours often share similar underlying mechanisms (i.e. impulsivity [1, 2]) and as such, predict similar negative outcomes including a disruption to educational attainment [3], poor physical [4] and mental health [5], and criminality persisting into adulthood [6]. Due to the cascade of negative outcomes associated with adolescent delinquency, identifying associated risk factors is crucial.

Of the many risk factors for adolescent delinquency, two are postulated to have a complex combined effect: childhood conduct problems and head injuries. Childhood conduct problems refer to violations of age-appropriate societal norms [7] and are associated with delinquent behaviour in adolescence [8–10] and adulthood [11, 12]. Furthermore, head injuries may similarly be associated with later delinquent behaviour [13–16]. Here, we refer to general head injuries which may result in seeking medical attention but do not result in ongoing impairment (i.e. a traumatic brain injury). However, evidence investigating the role of head injury on delinquency is limited and must be interpreted with caution. Notably, Mongilio and colleagues did not control for the potential influence neurodevelopmental disorders, such as conduct disorder [17], could have on this association [13], whilst Schwartz and colleagues suggested that the association between head injury and delinquency may be mediated by a relevant symptom of conduct disorder: impulse control [15]. Thus, whilst the literature alludes to an association between head injury and later delinquency, further evidence is required.

Head injuries and conduct problems may also share a bidirectional relationship that poses an additional risk for adolescent delinquency [18]. That is, childhood conduct problems are associated with an increased risk of head injuries and vice versa [18]. We hypothesize that when both conditions co-occur some of their underlying mechanisms (i.e. increased impulsivity [19, 20]) may create an additive effect, which will subsequently lead to an even greater risk for delinquency. However, no study has yet investigated the potential for a heightened risk of delinquent behaviour as a consequence of head injuries and conduct disorder occurring separately as well as in addition to one another. In response, this study analysed data from a large UK population-based birth cohort study to identify if the co-occurrence of childhood high levels of conduct problem symptoms and sustained head injuries posed a greater risk factor for adolescent delinquent behaviour at ages 14 and 17.

## Method

### Study design and participants

Participants were enrolled in the UK Millennium Cohort Study, a longitudinal birth cohort study following 18,786 individuals born between 2000 and 2002. They were measured at seven time points from time point 1 (T1) at age 9 months to time point 7 (T7) at age 17 years [21].

**Fig. 1:**
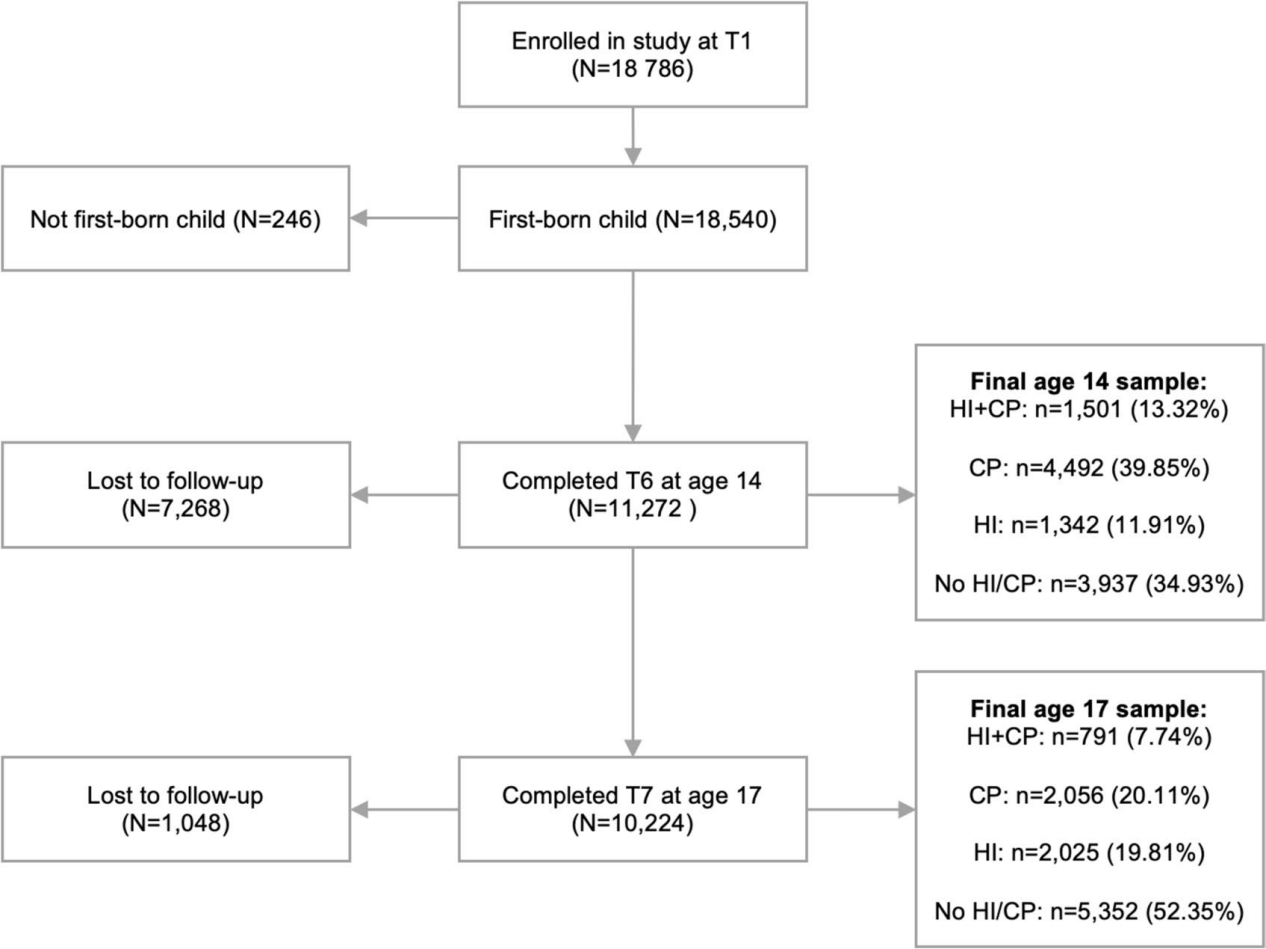
A flowchart of sample acquisition. This figure shows the exclusions made to reach the analytical samples and the breakdown of their groups. HI + CP refers to the group with a history of both high conduct problem symptoms and reported head injuries. CP refers to the group with a history of high conduct problem symptoms, but no reported head injuries. HI refers to the group with a history of reported head injury but no high levels of conduct problem symptoms. No HI/CP refers to the group without a history of either high conduct problems symptoms or reported head injuries

Two analytic samples were defined as participants enrolled at T1 and still enrolled at either age 14 or 17 years. Figure 1 presents an overview of sample selection. Only first-born children were included to allow independence of observation [22] and due to different levels of aggression and head injury risk between siblings [23].

Participants gave written consent for their data to be shared for secondary analysis. Ethical approval for this analysis was given by the University of Southampton Ethics Committee (ID = 62100.A1). The current study follows the appropriate Strengthening the Reporting of Observational Studies in Epidemiology (STROBE) reporting guidelines.

### Measures

#### Conduct problem (CP) symptoms

Conduct problem symptoms were measured using the Strength and Difficulties Questionnaire (SDQ) conduct problem subscale [24], a validated measure of conduct problems [25, 26] shown to be invariant across time points [27]. This subscale includes five items measured from 0 (‘not true’) to 2 (‘certainly true’), which are summed to produce a conduct problem symptom score (maximum score of 10). High conduct problem symptoms were determined by a score greater than 3 (see https://www.sdqinfo.org/).

#### Head injuries (HI)

Head injuries were parent reported during parent interviews. At each time point parents were asked if, since the last wave, their child had encountered an accident or injury which resulted in seeking a health professional. Those injuries coded as a bang to the head with or without a loss of consciousness were grouped to create a binary head injury variable (1 = present, 0 = absent) mirroring the classification used within the relevant literature [13].

#### Group classification

Four groups were created in each of the two analytic samples (estimating delinquency at ages 14 and 17) based on participant’s history of conduct problem symptoms and/or reported head injury.

The head injury and high conduct problem symptom (HI + CP) group had a history of high conduct problem symptoms (SDQ score > 3) and at least one reported head injury. The conduct problem (CP) group reported high conduct problem symptoms (SDQ score > 3), but not head injuries. The head injury (HI) group reported at least one head injury, but not high conduct problem symptoms. The fourth group reported no history of head injuries or high conduct problem symptoms (no HI/CP).

For age 14 delinquency, groups were determined by head injury and conduct problem data measured from T2 (age 3) to T5 (age 11). For age 17 delinquency, they were determined by head injury data measured from T2 to T6 (age 14) and by conduct problem data measured from T5 to T6. For further details on these groups and details of supplementary group classifications see Supplement 1.

Figure 1 displays the breakdown of groups for the age 14 and 17 analytical samples.

#### Adolescent delinquency

Adolescent delinquency was measured at ages 14 and 17 from nine items across substance use, criminality, and antisocial behaviour (see Supplement 2, Figure S1, and Table S1). An overall delinquency score at each age summed the nine delinquent behaviours (range 0–9) as is often created in the literature [13, 28]. A higher score indicated greater cumulative delinquency. Delinquency was further measured at each sub-level by summing relevant items, all of which had been recoded into binary (1 = yes, 0 = no) variables.

***Substance use.*** Participants self-reported smoking, binge drinking (> = 5 alcoholic drinks in one sitting), or using cannabis in the last 12 months. Substance use ranged from 0 to 3.

***Criminality.*** Participants self-reported ever being stopped or given a caution or formal warning by the police. At age 17, new variables were created to account for criminality reported since the last wave (age 14). Criminality scores ranged from 0 to 2.

***Antisocial behaviour***. Participants self-reported spray painting, damaging property, shoplifting, or stealing from someone else in the last 12 months. Antisocial behaviour scores ranged from 0 to 4.

#### Covariates

Study covariates included prenatal and socio-economic status (SES) risk factors all parent-reported at T1. We further included child sex, negative parenting styles, and ADHD (see Supplement 3). These covariates are commonly controlled for in delinquency research [8, 29], are associated with conduct problems [30, 31], or sustaining a head injury [32].

### Statistical analysis

All analyses were conducted in Stata, version 16.1 [33]. Missing data were present across various predictor and outcome variables and was accounted for using multiple imputation with chained equations (Stata’s MICE command), using 30 imputations. MCS survey weights at age 14 (T6) and 17 (T7), respectively, were included in the imputations and in all subsequent analyses to account for attrition, nonresponse bias, and stratification (more information on MCS weights can be found here: https://cls.ucl.ac.uk/wp-content/uploads/2017/07/User-Guide-to-Analysing-MCS-Data-using-Stata.pdf).

First, we tested the associations between childhood conduct problem symptoms and head injury status with age 14 and 17 delinquency (overall, substance use, criminality, and antisocial behaviour) using negative binomial regression models. All regression models included the aforementioned covariates. Supplementary regression models included conduct problem symptoms irrespective of head injury status and vice versa including head injuries which incurred a loss of consciousness only compared to those without a history of head injury.

## Results

Of 18,786 original participants, 11,272 were included in the age 14 analysis (5631 (50%) female and 9326 (82.7%) White) and 10,224 in the age 17 analysis (5107 (50%) female and 8349 (81.7%) White). Characteristics of the study populations and comparisons to the excluded samples are shown in Table 1. Though there were significant differences between some exposure and outcome variables these were weak (Cohen’s *d* < 0.23 or Cramér’s V < 0.11).

**Table 1:** Sample characteristics and differences between the analytical and excluded sample

Characteristics	Age 14 analytical sample		Age 14 excluded sample					Age 17 analytical sample		Age 17 excluded sample				
	N (%)	Mean (SD)	N (%)	Mean (SD)	Chi-Square (df)	*p* value	Cramer’s V	N (%)	Mean (SD)	N (%)	Mean (SD)	Chi-square (df)	*p* value	Cramér’s V
**Sex**					17.45 (1)	< 0.001	0.03					13.79 (1)	< 0.001	0.03
Male	5641 (50)	NA	3994 (53.2)	NA	NA	NA	NA	5117 (49.8)	NA	4518 (52.8)	NA	NA	NA	NA
Female	5631 (50)	NA	3520 (46.8)	NA	NA	NA	NA	5107 (50.2)	NA	4044 (47.2)	NA	NA	NA	NA
**Ethnicity**					43.27 (5)	< 0.001	0.05					45.98 (5)	< 0.001	0.05
White	9326 (82.7)	NA	6165 (82)	NA	NA	NA	NA	8349 (81.7)	NA	7142 (83.4)	NA	NA	NA	NA
Mixed	307 (2.7)	NA	255 (3.4)	NA	NA	NA	NA	297 (3.0)	NA	265 (3.1)	NA	NA	NA	NA
Black	353 (3.1)	NA	325 (4.3)	NA	NA	NA	NA	348 (3.1)	NA	330 (3.9)	NA	NA	NA	NA
Indian	292 (2.6)	NA	178 (2.4)	NA	NA	NA	NA	282 (2.6)	NA	188 (2.2)	NA	NA	NA	NA
Pakistani	836 (7.4)	NA	435 (5.8)	NA	NA	NA	NA	796 (7)	NA	475 (5.5)	NA	NA	NA	NA
Other	158 (1.4)	NA	108 (1.4)	NA	NA	NA	NA	152 (1.4)	NA	114 (1.3)	NA	NA	NA	NA
**Conduct problems**														
Age 3	9866 (87.5)	2.75 (2.03)	4492 (59.8)	2.95 (2.13)	5.25 (14,356)^a^	< 0.001	0.10^b^	8926 (87.3)	2.72 (2.02)	5432 (63.4)	2.96 (2.12)	6.70 (14,356)^a^	< 0.001	0.12^b^
Age 5	10,324 (91.6)	1.45 (1.47)	4069 (54.2)	1.64 (1.60)	6.64 (14,391)^a^	< 0.001	0.12^b^	9354 (91.5)	1.44 (1.47)	5039 (58.9)	1.63 (1.58)	7.13 (14,391)^a^	< 0.001	0.13^b^
Age 7	10,115 (89.7)	1.33 (1.51)	3,035 (40.4)	1.58 (1.66)	7.89 (13,148)^a^	< .001	0.16^b^	9167 (89.7)	1.31 (1.49)	3983 (46.5)	1.56 (1.66)	8.70 (13,148)^a^	< 0.001	0.17^b^
Age 11	10,366 (92.0)	1.34 (1.54)	2035 (27.1)	1.61 (1.70)	7.26 (12,399)^a^	< 0.001	0.18^b^	9341 (91.4)	1.30 (1.51)	3060 (35.7)	1.63 (1.72)	10.30 (12,399)^a^	< 0.001	0.22^b^
Age 14	NA	NA	NA	NA	NA	NA	NA	9113 (89.1)	1.35 (1.59)	1944 (22.7)	1.63 (1.74)	6.84 (11,055)^a^	< 0.001	0.17^b^
**Head injuries**														
9 months–3 years	1289 (11.4)	NA	596 (7.9)	NA	60.90 (1)	< 0.001	0.06	929 (9.1)	NA	576 (6.7)	NA	35.19 (1)	< 0.001	0.04
3–5 years	952 (8.4)	NA	452 (6.0)	NA	38.23 (1)	< 0.001	0.05	701 (6.9)	NA	422 (4.9)	NA	30.81 (1)	< 0.001	0.04
5–7 years	720 (6.4)	NA	243 (3.2)	NA	91.85 (1)	< 0.001	0.07	515 (5.0)	NA	264 (3.1)	NA	44.76 (1)	< 0.001	0.05
7–11 years	616 (5.5)	NA	160 (2.1)	NA	126.32 (1)	< 0.001	0.08	444 (4.3)	NA	176 (2.1)	NA	76.37 (1)	< 0.001	0.06
11–14 years	NA	NA	NA	NA	NA	NA	NA	359 (3.5)	NA	56 (0.1)	NA	176.10 (1)	< 0.001	0.10
**Delinquency**^**c**^														
Binge drinking	940 (8.3)	NA	9 (0.1)	NA	0.25 (1)	0.620	0.02	4648 (48.8)	NA	166 (1.9)	NA	0.19 (1)	0.666	0.01
Smoking	387 (3.4)	NA	4 (0.1)	NA	0.48 (1)	0.487	0.01	1863 (18.2)	NA	63 (0.7)	NA	0.01 (1)	0.931	0.001
Cannabis use	470 (4.2)	NA	3 (< .01)	NA	2.25 (1)	0.133	0.01	2356 (23.0)	NA	85 (1.0)	NA	0.31 (1)	0.577	0.01
Stopped by police	1479 (13.1)	NA	18 (0.2)	NA	0.63 (1)	0.427	0.01	1124 (11.0)	NA	10 (0.1)	NA	1.71 (1)	0.191	0.02
Cautioned	790 (7.0)	NA	10 (0.1)	NA	0.20 (1)	0.658	0.004	108 (1.1)	NA	3 (< .01)	NA	10.85 (1)	< 0.001	0.03
Spray paint	284 (2.5)	NA	5 (.1)	NA	0.19 (1)	0.662	0.004	276 (2.7)	NA	11 (0.1)	NA	0.32 (1)	0.572	0.01
Property damage	333 (3.0)	NA	5 (0.1)	NA	0.01 (1)	0.937	0.001	302 (3.0)	NA	7 (0.1)	NA	1.02 (1)	0.313	0.01
Shoplifting	357 (3.2)	NA	3 (< .01)	NA	0.94 (1)	0.333	0.01	536 (5.2)	NA	18 (0.2)	NA	< 0.001 (1)	0.989	< 0.001
Stealing	121 (1.1)	NA	3 (< .01)	NA	.87 (1)	.350	.01	149 (1.5)	NA	6 (0.1)	NA	0.19 (1)	.667	.004

If (*n*) is less than the *n* included, this refers to missing data within the variable

^a^Independent samples *t* test

^b^Cohen’s *d*

^c^For age 14 and 17 analytical samples, delinquency as measured at age 14 and 17, respectively

**Fig. 2:**
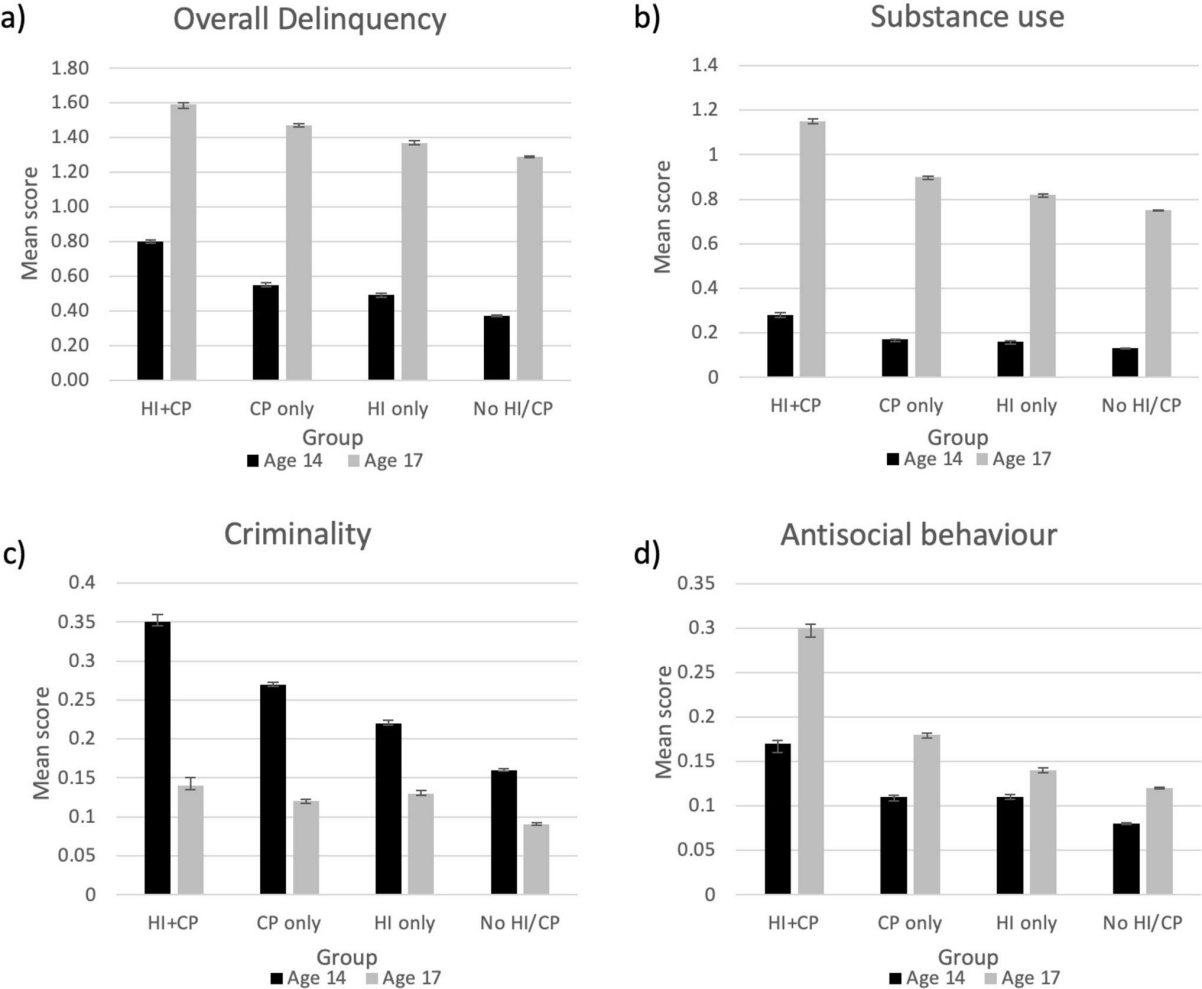
The mean delinquency scores of groups defined by childhood conduct problem symptom and head injury status. This figure shows the mean scores of (**a**) overall delinquency, (**b**) substance use, (**c**) criminality, and (**d**) antisocial behaviour at ages 14 (black) and 17 (grey). These scores are displayed for (1) those with no history of high conduct problem symptoms or head injuries (HI + CP), (2) those with a history only of high conduct problem symptoms and not head injury (CP only), (3) those with a history of sustaining a head injury but no high levels of conduct problem symptoms (HI only), and (4) those without a history of both high conduct problems and sustaining a head injury (no HI/CP). This figure shows mean scores typically increasing from ages 14 to 17 with the exception of criminality

Figure 2 displays the levels of (a) overall delinquency, (b) substance use, (c) criminality, and (d) antisocial behaviour reported by each group. Levels of delinquency generally increased from ages 14 to 17 with the exception of criminality. At ages 14 and 17, mean levels of conduct problem symptoms between the HI + CP and CP groups (*t*(4,399) = −1.55, *p* = 0.120; *t*(1,438) = −1.09, *p* = 0.279, respectively) and mean rates of head injury between the HI + CP and HI groups (*t*(2,842) = −0.06, *p* = 0.520; *t*(2,815) = −0.26, *p* = 0.798, respectively) did not significantly differ.

### Age 14 delinquency

At age 14, 2489 (22.08%) participants reported at least one delinquent behaviour. A summary of the regression models is shown in Table 2. The HI + CP and CP groups were associated with a significantly greater risk of reporting overall delinquency, substance use, criminality, and antisocial behaviour compared to the no HI/CP group. The HI + CP group was also associated with a significantly greater risk of overall delinquency and substance use when compared to the CP and HI groups.

**Table 2:** Adolescent cumulative delinquency at age 14 predicted by childhood conduct problems and head injury during ages 3 to 11

	Overall delinquency	Substance use	Crime	Antisocial behaviour
	IRR (95% CI)	IRR (95% CI)	IRR (95% CI)	IRR (95% CI)
HI + CP vs no HI/CP	1.60** (1.34–1.91)	1.80** (1.40–2.31)	1.56** (1.29–1.90)	1.41* (1.04–1.91)
HI + CP vs CP	1.20* (1.01–1.43)	1.38* (1.08–1.77)	1.12 (0.94–1.33)	1.13 (0.85–1.51)
HI + CP vs HI	1.39* (1.13–1.72)	1.48* (1.11–1.98)	1.52** (1.21–1.92)	1.11 (0.78–1.59)
CP vs no HI/CP	1.33* (1.13–1.56)	1.30* (1.05–1.62)	1.40** (1.17–1.25)	1.24 (0.94–1.64)
CP vs HI	1.16 (0.96–1.39)	1.07 (0.83–1.38)	1.36* (1.12–1.66)	0.98 (0.71–1.36)
HI vs no HI/CP	1.15 (0.96–1.37)	1.21 (0.97–1.52)	1.03 (0.84–1.25)	1.27 (0.95–1.69)
Sex	1.22** (1.09–1.36)	0.78* (0.67–0.90)	1.54** (1.37–1.74)	1.47** (1.21–1.78)
ADHD	0.95 (0.69–1.30)	0.74 (0.48–1.16)	0.96 (0.70–1.32)	1.20 (0.76–1.92)
Low birth weight	0.76 (0.59–0.99)	0.58* (0.38–0.86)	0.98 (0.73–1.32)	0.74 (0.47–1.17)
Premature birth	0.98 (0.74–1.28)	1.09 (0.72–1.65)	0.90 (0.65–1.24)	0.81 (0.51–1.27)
Smoking during pregnancy	1.84** (1.62–2.08)	2.15** (1.81–2.57)	1.79** (1.57–2.03)	1.44* (1.16–1.78)
Alcohol during pregnancy	1.14* (1.02–1.29)	1.35** (1.16–1.58)	1.01 (0.89–1.14)	1.08 (0.88–1.33)
Teenage pregnancy	1.34 (0.97–1.85)	1.37 (0.87–2.14)	1.33 (0.99–1.77)	1.07 (0.63–1.80)
Low parental education	0.92 (0.77–1.09)	0.87 (0.68–1.12)	0.95 (0.80–1.13)	0.89 (0.65–1.22)
Low parent occupation	1.18* (1.03–1.36)	1.02 (0.84–1.24)	1.32** (1.13–1.54)	1.20 (0.96–1.51)
Low household income	1.14 (0.99–1.32)	1.07 (0.88–1.30)	1.23* (1.05–1.43)	1.08 (0.83–1.39)
Single parent household	0.87 (0.74–1.02)	0.89 (0.70–1.13)	0.91 (0.77–1.07)	0.80 (0.61–1.06)
Harsh parenting	1.02 (0.99–1.06)	1.04 (1.00–1.09)	1.00 (0.97–1.03)	1.05 (1.00–1.10)
Parental withdrawal tactics	1.00 (0.98–1.03)	0.99 (0.95–1.02)	1.01 (0.99–1.04)	0.99 (0.95–1.03)

X vs Y, Y is the reference group

*IRR* incidence rate ratio, *HI* head injury, *CP* conduct problem symptoms

*p < 0.05

**p < 0.001

We found no evidence of an association between the HI group and any delinquent behaviour (Table 2). However, post hoc analyses identified a significant association between a history of head injuries (irrespective of conduct problem symptoms) with overall delinquency and substance use compared to a group with no history of head injury (see Supplementary Table S2). This association remained significant but became stronger when a loss of consciousness was compared to those without a history of head injury (see Supplementary Table S2). Further associations were identified between conduct problems (irrespective of head injury) with overall delinquency, substance use, and crime compared to a group without a history of conduct problems (see Supplementary Table S2).

### Age 17 delinquency

At age 17, 5,461 (53.41%) participants reported at least one delinquent behaviour. A complete summary of the regression models is shown in Table 3. Compared to the no HI/CP group, the HI + CP and CP groups showed an increased rate of overall delinquency whilst the HI + CP group also showed further increased rates of substance use. Both the HI + CP and CP groups also showed significantly increased rates of antisocial behaviour compared to the no HI/CP and HI groups. There was no evidence for increased rates of criminality in any of the groups nor any significant differences between the HI + CP and CP groups.

**Table 3:** Adolescent cumulative delinquency at age 17 predicted by childhood conduct problems at ages 11 and 14 and head injury during ages 3 to 14

	Overall delinquency	Substance use	Criminality	Antisocial behaviour
	IRR (95% CI)	IRR (95% CI)	IRR (95% CI)	IRR (95% CI)
HI + CP vs no HI/CP	1.33* (1.08–1.65)	1.32* (1.07–1.64)	1.11 (0.63–1.93)	1.55* (1.01–2.36)
HI + CP vs CP	1.08 (0.83–1.41)	1.14 (0.87–1.48)	0.93 (0.46–1.86)	0.92 (0.52–1.62)
HI + CP vs HI	1.27 (1.00–1.60)	1.27* (1.01–1.60)	0.87 (0.49–1.53)	1.58* (1.00–2.47)
CP vs no HI/CP	1.23* (1.02–1.49)	1.16 (0.97–1.40)	1.19 (0.68–2.07)	1.69* (1.02–2.79)
CP vs HI	1.17 (0.96–1.43)	1.12 (0.92–1.35)	0.93 (0.56–1.55)	1.72* (1.07–2.77)
HI vs no HI/CP	1.05 (0.95–1.17)	1.04 (0.94–1.15)	1.28 (0.97–1.67)	0.98 (0.76–1.27)
Sex	1.21** (1.10–1.33)	1.11* (1.01–1.21)	1.37* (1.04–1.81)	2.01** (1.54–2.62)
ADHD	1.08 (0.84–1.40)	1.05 (0.80–1.38)	1.27 (0.65–2.49)	1.11 (0.68–1.81)
Low birth weight	0.89 (0.66–1.21)	0.88 (0.61–1.27)	0.95 (0.57–1.60)	0.92 (0.51–1.66)
Premature birth	1.00 (0.76–1.30)	1.10 (0.81–1.47)	0.62 (0.34–1.14)	0.75 (0.41–1.38)
Smoking during pregnancy	1.30** (1.15–1.47)	1.34** (1.19–1.51)	1.01 (0.71–1.44)	1.28 (0.91–1.80)
Alcohol during pregnancy	1.24** (1.14–1.35)	1.30** (1.19–1.42)	0.96 (0.75–1.24)	1.07 (0.85–1.35)
Teenage pregnancy	0.99 (0.69–1.43)	1.16 (0.79–1.71)	0.61 (0.26–1.48)	0.37 (0.14–1.03)
Low parental education	0.82 (0.67–1.02)	0.80* (0.65–0.99)	0.97 (0.54–1.77)	0.93 (0.52–1.67)
Low parental occupation	0.89 (0.78–1.01)	0.86* (0.76–0.98)	1.06 (0.77–1.45)	0.95 (0.69–1.31)
Low household income	0.85* (0.73–0.98)	0.83* (0.72–0.95)	1.27 (0.90–1.80)	0.69 (0.46–1.03)
Single parent household	0.96 (0.80–1.14)	1.00 (0.83–1.19)	1.12 (70–1.77)	0.70 (0.44–1.10)
Harsh parenting	1.03 (1.00–1.05)	1.02 (0.99–1.05)	1.09* (1.01–1.19)	1.02 (0.94–1.11)
Parental withdrawal tactics	1.01 (0.99–1.04)	1.02 (0.99–1.04)	1.00 (0.94–1.07)	1.00 (0.94–1.08)

X vs Y, Y is the reference group

*IRR* incidence rate ratio, *HI* head injury, *CP* conduct problem symptoms

*p < 0.05

**p < 0.001

Post hoc analyses found further evidence for a significant association between a history of conduct problems (irrespective of head injury status) with overall delinquency and substance use compared to those without a history of conduct problem symptoms (see Supplementary Table S3). There was no evidence for an increased rate of delinquency in those with a head injury (irrespective of conduct problem symptom status) even when considering those head injuries with a loss of consciousness only (see Supplementary Table S3).

## Discussion

This large, prospective cohort study provides novel evidence for a greater risk of early delinquency following the co-occurrence of childhood head injuries and high conduct problem symptoms compared to a history of one or neither, when controlling for common risk factors. This is the first study to show that this co-occurrence is associated with an earlier increased risk of delinquency by age 14 compared to all other groups.

In line with previous studies [8–10], childhood conduct problems were associated with an increased risk of earlier delinquency compared to those without the presence of either at age 14, and this was significantly greater when accompanied by co-occurring head injuries. This may be explained by the bidirectional association between childhood conduct problems and head injuries across development [18], which may exacerbate one another’s characteristics associated with subsequent delinquency (i.e. increased impulsivity). That is, causal models of conduct disorder argue that environmental factors, such as childhood adversity, can result in altered cognitive and neural functioning (i.e. poor executive functioning or hypervigilance to aggressive cues) and this can increase the risk of conduct problem symptoms [34] and engagement in delinquent behaviours [1]. Similarly, common cognitive impairments following head injuries relating to emotional, behavioural, and social difficulties [35], including impulsivity [19], may further increase such engagement in delinquent behaviours [15]. When head injuries and high conduct problem symptoms co-occur in middle childhood, these respective impairments may thus add up or interact to result in a significantly greater risk of early adolescent delinquency.

In contrast, our findings show that childhood head injuries without co-occurring high conduct problem symptoms do not predict adolescent delinquency. This suggests that the neural and cognitive impairments associated with childhood head injury may be modestly associated with adolescent delinquency. Only when they present alongside co-occurring high conduct problem symptoms and generate an accumulative or additive effect do the impairments then create a significantly increased risk of adolescent delinquency. This contradicts the findings reported in the literature, which suggest head injury is associated with various forms of delinquency [13–16]. The disparity may arise from our investigating head injuries whilst excluding co-occurring conduct problem symptoms. Only when we considered a history of head injury regardless of (as was done in the previous literature) or explicitly alongside high conduct problem symptoms, did we identify an association with early overall delinquency and substance use. As the previous literature did not account for conduct problem symptoms, it is likely that their findings are confounded by similarly high levels of conduct problems.

In alignment with previous research [8–11], we found evidence for an increased rate of age 17 delinquency (overall, substance use, and antisocial behaviour) following conduct problem symptoms regardless of head injury history. That is, childhood head injuries did not exacerbate the association above and beyond when later conduct problem symptoms were present without a history of head injury. With rates of reported head injuries decreasing throughout development, we argue that their additive effect alongside co-occurring conduct problem symptoms may dissipate overtime leaving only later conduct problem symptoms (as measured at ages 11 and 14) to drive continued delinquency.

Notably, the findings showed no evidence for an association between conduct problem symptoms and later criminality at age 17. This could be due to a general decrease in criminality across all groups. Such a decrease in criminality may be explained by delayed maturation of the cognitive control systems as described by the dual systems theory [36]. An early mismatch in the maturation of the cognitive control system and the socioemotional system may make adolescents vulnerable to delinquent behaviour. By late adolescents, the systems converge allowing the cognitive control system to effectively provide top-down control to override illegal behaviours.

### Strength and limitations

This study used a prospective birth cohort study and therefore the results are generalizable to children born in the UK between 2000 and 2002. Its longitudinal design is a further strength. By analysing such data, this is the first study to identify the longitudinal nature of the associations between childhood conduct problems and head injuries and subsequent adolescent delinquency.

This study does present with limitations, however. Conduct problems were measured using the SDQ, which is not a diagnostic measure. As such, we note the continued reference to ‘conduct problem symptoms’ throughout. The groups created in this study also homogenise head injury. That is, it includes those with a history of a single or ‘one-off’ head injury as well as those with multiple head injuries. Further analysis on larger datasets that can differentiate between the number of head injuries is required to identify how this may impact on delinquency. In addition, we cannot infer causation because the criminality measures were not temporally limited to after the reporting of conduct problem symptoms and head injury. We endeavoured to minimise this by creating variables at age 17 which accounted only for criminality reported after age 14. However, the age 14 criminality variables remain problematic with the potential of reverse causation. This must be taken into consideration when interpreting the results.

## Conclusions

This study provides novel findings which add to our understanding of how early adolescent delinquency may occur. Importantly, it suggests that sustaining a mild head injury during childhood without exhibiting conduct problem symptoms may not increase one’s risk of adolescent delinquency. Nonetheless, if they co-occur alongside higher levels of conduct problem symptoms, there appears to be an increased risk on early adolescent delinquency above and beyond when they occur separately or not at all. These novel findings are important in furthering our understanding of adolescent delinquency whilst highlighting the potential negative implications of childhood co-occurring head injuries and high conduct problem symptoms.

### Supplementary Information

Below is the link to the electronic supplementary material.

Supplementary file1 (DOCX 3233 KB)

## Data Availability

The MCS dataset used in this study is freely available via the UK Data Service Archive (beta.ukdataservice.ac.uk/datacatalogue/series/series?id=2000031).
