# Supplementary material for: Adolescent delinquency following co-occurring childhood head injuries and conduct problem symptoms: findings from a UK longitudinal birth cohort
Source: Eur Child Adolesc Psychiatry. 2023 Dec 28;33(8):2571–80. doi: 10.1007/s00787-023-02335-0 (PMC11272693; doi:10.1007/s00787-023-02335-0)
Supplement: Supplementary file 1 — Supplementary file1 (DOCX 3233 KB) [file 787_2023_2335_MOESM1_ESM.docx]

**Supplementary Material**

Table of Contents

[Supplement 1 2](#_Toc148042828)

[Supplement 2 3](#_Toc148042829)

[Figure S1. Scree Plots Highlighting the Appropriate Number of Factors for Delinquency at a) 14 and b) 17 Years 3](#_Toc148042830)

[Table S1. Pattern Matrix of the Factor Loadings for a Three-factor Solution at Ages 14 and 17 3](#_Toc148042831)

[Supplement 3 4](#_Toc148042832)

[Table S2. Adolescent Cumulative Delinquency at Age 14 Predicted by Childhood Conduct Problems, a Bang to the Head, or a Loss of Consciousness during ages 3 to 11 5](#_Toc148042833)

[Table S3. Adolescent Cumulative Delinquency at Age 17 Predicted by Childhood Conduct Problems from ages 11 and 14, or a Bang to the Head or a Loss of Consciousness during ages 3 to 14 6](#_Toc148042834)

[References 7](#_Toc148042835)

# **Supplement 1**

There are different trajectories or sub-types of conduct problems [1], even when accounting for their association with head injury [2]. These sub-types include a child-limited (symptoms present only in early childhood, i.e., <11 years old), persistent (present across childhood and into adolescents), and adolescent-onset (present from age 11 onwards). However, a recent meta-analysis suggests that whilst persistent and adolescent-onset conduct problems are significant predictors of later delinquency, earlier levels of conduct problems (i.e., childhood-limited) show weak or non-significant associations [3]. Thus, whilst our age 14 analyses consider conduct problem symptoms across development, we have limited our group classifications to conduct problem symptoms measured at ages 11 or 14 only for age 17 delinquency.

We created further supplementary groups, which aimed to replicate previously identified associations between delinquency and conduct problem symptoms or head injuries separately (i.e., without taking the presence of the other into consideration) [4-7].

We further specified a head injury group consisting of those who sustained a head injury with a loss of consciousness only. As this is a much smaller subsample, we had to compare this group to a random subsample as opposed to the entire MCS population without a history of head injury.

# **Supplement 2**

Whilst the three chosen sub-categories of delinquency were guided by previous literature [7,8], an Exploratory Factor Analysis with a direct oblimin rotation was computed to ensure that these categories were appropriate. The scree plots below show that a three-factor solution was the most appropriate at ages 14 and 17 (Figure S1). Results of this solution are further shown in the pattern matrices below (Table S1).

# **Figure S1. Scree Plots Highlighting the Appropriate Number of Factors from an Exploratory Factor Analysis for Delinquency at a) 14 and b) 17 Years**

**
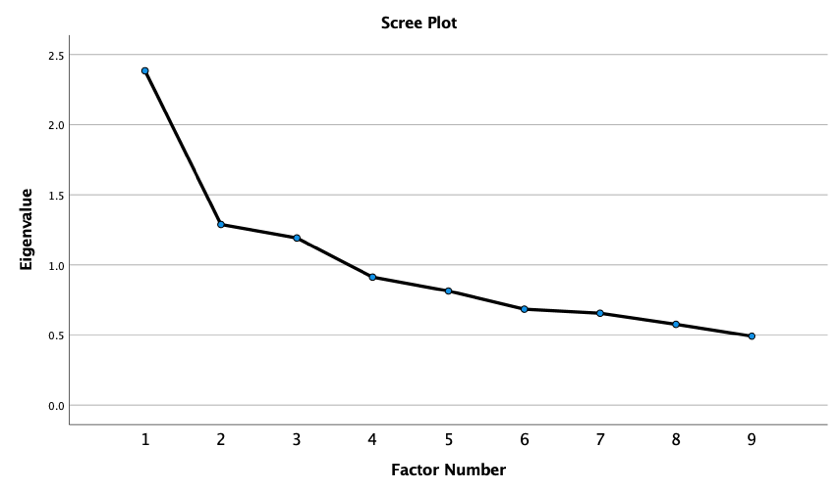
**
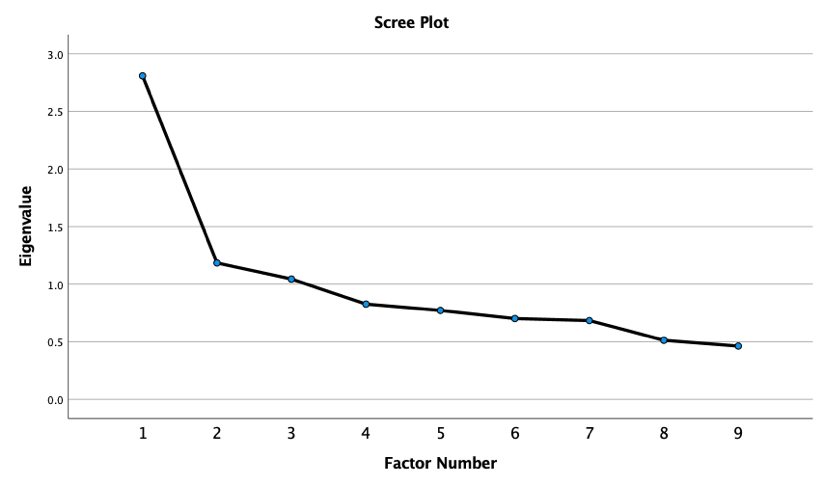


b)

a)

This figure highlights the scree plots produced from a factor analysis of the delinquency items at ages 14 (a) and 17 (b). they show that at both ages, a three-factor solution appears to be appropriate.

# **Table S1. Pattern Matrix of the Factor Loadings for an Exploratory Factor Analysis with a Three-factor Solution at Ages 14 and 17**

|  | **Age 14 Factors** | | |  | **Age 17 Factors** | | |
| --- | --- | --- | --- | --- | --- | --- | --- |
| **Items** | **Substance use** | **Antisocial behaviour** | **Crime** |  | **Substance use** | **Antisocial behaviour** | **Crime** |
| Smoking | .69 |  |  |  | .61 |  |  |
| Cannabis use | .79 |  |  |  | .77 |  |  |
| Binge drinking | .45 |  |  |  | .52 |  |  |
| Steal |  | .45 |  |  |  | .39 |  |
| Shoplift |  | .31 |  |  |  | .45 |  |
| Weapon |  | .44 |  |  |  | .31 |  |
| Property damage |  | .31 |  |  |  | .58 |  |
| Stopped by police |  |  | .75 |  |  |  | .64 |
| Cautioned by police |  |  | .63 |  |  |  | .65 |

# **Supplement 3**

Prenatal covariates included low birth weight (<2.5kg [9,10]), premature birth (<=252 days [9,10]), and mother smoking and drinking during pregnancy [11].

SES covariates included parental education level (not achieved a high school diploma/GCSE’s [11,12]), parental occupation status (semi-skilled or less [13]), single parent household [12,14], low household income (below 60% median poverty indicator [12,14]), and teenage pregnancy (<18 years-old [12]).

Negative parenting styles were measured using Straus’s Conflict Tactic Scale at T2 [15] and encompasses harsh parenting (smacking, shouting at, or telling off the child) and parental withdrawal tactics (ignoring child, sending them to their room, and taking away their toys). All items were measured on a 5-point Likert scale to ascertain the frequency of the behaviours (1 = never, 5=daily) and were summed to create an overall harsh parenting (range 3 – 15) and an overall withdrawal tactics score (range 3-15). A higher scored indicated harsher parenting or greater use of withdrawal tactics.

ADHD was measured by parent-reports of an ADHD diagnosis for their child. This was asked from T3 to T6 (age 5 to 14) and was summarized via a binary variable (0= no diagnosis, 1 = ADHD diagnosis).

# **Table S2. Adolescent Cumulative Delinquency at Age 14 Predicted by Childhood Conduct Problems, a Bang to the Head, or a Loss of Consciousness during ages 3 to 11**

|  | **Overall delinquency** | **Substance use** | **Crime** | **Antisocial behaviour** |
| --- | --- | --- | --- | --- |
|  | **IRR (95% CI)** | **IRR (95% CI)** | **IRR (95% CI)** | **IRR (95% CI)** |
| HI vs no HI | **1.19* (1.06 – 1.34)** | **1.28* (1.08 - 1.50)** | 1.07 (.94 - 1.21) | 1.19 (.97 - 1.46) |
| LoC vs no HI^a^ | **2.04* (1.25 - 3.31)** | **4.27** (1.80 - 10.15)** | 1.70 (.85 - 3.42) | 1.10 (.51 – 2.37) |
| CP vs no CP | **1.35** (1.18 - 1.55)** | **1.37* (1.14 - 1.65)** | **1.44** (1.25 - 1.67)** | 1.21 (.96 - 1.52) |

Abbreviations: IRR, incidence rate ratio; HI, head injury (a bang to the head with or without a loss of consciousness); LoC, loss of consciousness; CP, conduct problem symptoms

*Note.* X vs Y, Y is the reference group

^a^When using a random subsample of those without a history of head injury

**p*<.05

***p*<.001

# **Table S3. Adolescent Cumulative Delinquency at Age 17 Predicted by Childhood Conduct Problems from ages 11 and 14, or a Bang to the Head or a Loss of Consciousness during ages 3 to 14**

|  | **Overall delinquency** | **Substance use** | **Crime** | **Antisocial behaviour** |
| --- | --- | --- | --- | --- |
|  | **IRR (95% CI)** | **IRR (95% CI)** | **IRR (95% CI)** | **IRR (95% CI)** |
| HI vs no HI | 1.06 (.97 - 1.16) | 1.06 (.97 - 1.16) | 1.20 (.94 - 1.52) | .98 (.79 – 1.22) |
| LoC vs no HI | 1.12 (.88 - 1.42) | 1.10 (.87 - 1.38) | 1.88 (.90 - 3.89) | 1.48 (.60 - 3.64) |
| CP vs no CP | **1.24* (1.07 - 1.45)** | **1.20* (1.03 - 1.39)** | 1.09 (.71 - 1.67) | **1.65* (1.12 - 2.44)** |

Abbreviations: IRR, incidence rate ratio; HI, head injury (a bang to the head with or without a loss of consciousness); LoC, loss of consciousness; CP, conduct problem symptoms

*Note.* X vs Y, Y is the reference group

**p*<.05

***p*<.001

# **References**

1 Gutman LM, Joshi H, Schoon I. 2019. Developmental Trajectories of Conduct Problems and Cumulative Risk from Early Childhood to Adolescence. J Youth Adolesc 48(2):181-98. <https://doi.org/10.1007/s10964-018-0971-x>.

2 Carr HR, Brandt VC, Golm D, Hall JE. 2023. Linked head injury and conduct problem symptom pathways from early childhood to adolescence and their associated risks: Evidence from the millennium cohort study. Dev Psychopathol:1-9. <https://doi.org/10.1017/S0954579423001062>.

3 Bevilacqua L, Hale D, Barker ED, Viner R. 2018. Conduct problems trajectories and psychosocial outcomes: a systematic review and meta-analysis. European Child & Adolescent Psychiatry 27(10):1239-60. <https://doi.org/10.1007/s00787-017-1053-4>.

4 Mongilio J. 2022. Childhood Head Injury as an Acquired Neuropsychological Risk Factor for Adolescent Delinquency. Journal of Research in Crime and Delinquency 59(6):756-90. <https://doi.org/10.1177/00224278221081140>.

5 Hopfer C, Salomonsen-Sautel S, Mikulich-Gilbertson S, Min SJ, McQueen M, Crowley T, et al. 2013. Conduct disorder and initiation of substance use: a prospective longitudinal study. J Am Acad Child Adolesc Psychiatry 52(5):511-18.e4. <https://doi.org/10.1016/j.jaac.2013.02.014>.

6 Hammerton G, Murray J, Maughan B, Barros FC, Gonçalves H, Menezes AMB, et al. 2019. Childhood behavioural problems and adverse outcomes in early adulthood: A comparison of Brazilian and British birth cohorts. Journal of Developmental and Life-Course Criminology 5(4):517-35. <https://doi.org/10.1007/s40865-019-00126-3>.

7 Picoito J, Santos C, Nunes C. 2021. Emotional and behavioural pathways to adolescent substance use and antisocial behaviour: results from the UK Millennium Cohort Study. European Child & Adolescent Psychiatry 30(11):1813-23. <https://doi.org/10.1007/s00787-020-01661-x>.

8 Jackson DB, Testa A, Boccio CM. 2022. Police Stops and Adolescent Substance Use: Findings From the United Kingdom Millennium Cohort Study. Journal of Adolescent Health 70(2):305-12. <https://doi.org/https://doi.org/10.1016/j.jadohealth.2021.08.024>.

9 Reijneveld SA, de Kleine MJ, van Baar AL, Kollée LA, Verhaak CM, Verhulst FC, et al. 2006. Behavioural and emotional problems in very preterm and very low birthweight infants at age 5 years. Arch Dis Child Fetal Neonatal Ed 91(6):F423-8. <https://doi.org/10.1136/adc.2006.093674>.

10 Whiteside-Mansell L, Bradley RH, Casey PH, Fussell JJ, Conners-Burrow NA. 2009. Triple risk: do difficult temperament and family conflict increase the likelihood of behavioral maladjustment in children born low birth weight and preterm? J Pediatr Psychol 34(4):396-405. <https://doi.org/10.1093/jpepsy/jsn089>.

11 Van Adrichem DS, Huijbregts SCJ, Van Der Heijden KB, Van Goozen SHM, Swaab H. 2020. Aggressive behavior during toddlerhood: Interrelated effects of prenatal risk factors, negative affect, and cognition. Child Neuropsychology 26(7):982-1004. <https://doi.org/10.1080/09297049.2020.1769582>.

12 Trentacosta CJ, Hyde LW, Shaw DS, Dishion TJ, Gardner F, Wilson M. 2008. The relations among cumulative risk, parenting, and behavior problems during early childhood. J Child Psychol Psychiatry 49(11):1211-19. <https://doi.org/10.1111/j.1469-7610.2008.01941.x>.

13 Greitemeyer T, Sagioglou C. 2016. Subjective socioeconomic status causes aggression: A test of the theory of social deprivation. J Pers Soc Psychol 111(2):178-94. <https://doi.org/10.1037/pspi0000058>.

14 Northerner LM, Trentacosta CJ, McLear CM. 2016. Negative Affectivity Moderates Associations between Cumulative Risk and At-Risk Toddlers' Behavior Problems. J Child Fam Stud 25(2):691-99. <https://doi.org/10.1007/s10826-015-0248-x>.

15 Straus MA, Hamby SL, Finkelhor D, Moore DW, Runyan D. 1998. Identification of child maltreatment with the Parent-Child Conflict Tactics Scales: Development and psychometric data for a national sample of American parents. Child abuse & neglect 22(4):249-70. <https://doi.org/10.1016/s0145-2134(97)00174-9>.
